# A New Tile in the Biochemical Puzzle of Insulin Biology

**DOI:** 10.1371/journal.pbio.0040059

**Published:** 2006-01-31

**Authors:** Françoise Chanut

Molecular biologists are like obsessive jigsaw-puzzle lovers. They won't stop until they have found a spot for every molecule in their favorite cell. But their obsession rarely amounts to just fun and games, particularly when that cell is linked to a serious disease. By patiently fitting individual proteins into simple biochemical pathways, and biochemical pathways into complex cell functions, molecular biologists establish solid scaffolds to support effective therapies.

One such highly scrutinized cell is the pancreatic ß cell. These cells produce insulin, a key hormonal regulator of metabolism and blood sugar content. Their malfunction or premature loss causes type I—or juvenile onset—diabetes, which affects millions of people worldwide. Type I diabetics respond well to regular injections of synthetic insulin, but this lifelong treatment is not without shortcomings. Therapies based on ß cell replacement, using stem cells for instance, are promising but depend on engineering replacement cells that mimic ß cells closely, which require an in-depth understanding of ß cell biology.

ß cells store insulin and release it when needed: for instance, when post-meal glucose levels rise far above one gram per liter. A rapid return to normal blood sugar level ensues, because insulin stimulates glucose uptake by muscles and other tissues. But once insulin stores are depleted, they must be replenished, which the ß cell accomplishes by keeping its insulin gene active. The expression level of any gene usually reflects the combined influence of a variety of activating or inhibitory biochemical pathways. Cells use molecular signals to modulate the pathways' outputs according to their needs. In a new study in *PLoS Biology*, Nora Smart, Seung Kim, and their colleagues show that one of the pathways that regulates insulin gene expression in ß cells is the TGF-ß signaling cascade. Furthermore, they find that TGF-ß signaling is crucial for the maintenance of mature ß cell identity.

TGF-ß is the founding member of a large family of extracellular proteins that control a variety of cell decisions. All TGF-ß family members act by binding to a receptor in the cell membrane. This triggers an enzymatic cascade that adds phosphate tags to a series of intracellular proteins called Smads. Some Smads transmit the TGF-ß signal; others interrupt it by preventing the phosphorylation of activating Smads. Eventually, a phosphorylated activating Smad enters the cell's nucleus, where it controls the expression of various target genes. [Fig pbio-0040059-g001]

**Figure pbio-0040059-g001:**
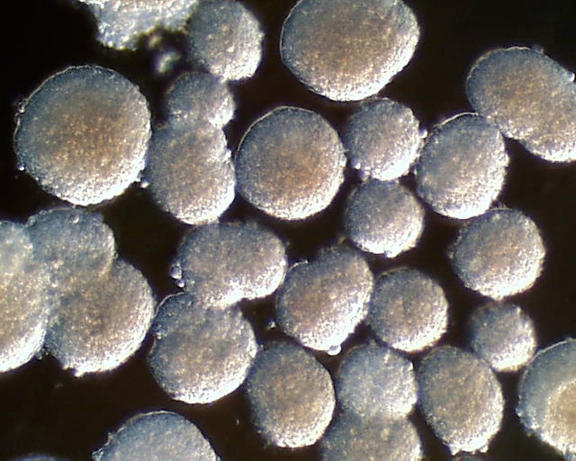
TGF-ß signaling is essential for maintaining the identity and function of pancreatic islet ß-cells. (Image: Hainan Chen and Seung Kim)

Work from several labs, including Kim's, has shown that TGF-ß signaling is important for the formation of ß cells within the developing pancreas. But mature, adult ß cells also display telltale signs of active TGF-ß signaling, such as phosphorylated Smads, which suggest that TGF-ß signaling could also control the normal functioning of pancreatic ß cells. Smart and her colleagues interrupted TGF-ß signaling in ß cells with one of the inhibitory Smad family members, Smad7. The difficulty was to express Smad7 in mature ß cells only, and not at earlier stages, when it would prevent ß cell formation. The researchers engineered mice with a modified Smad7 gene (transgene) that expressed Smad7 specifically in ß cells and could be turned off at will with the drug doxycycline. By simply adding or eliminating doxycycline in the animals' diet, the researchers controlled the timing of Smad7 expression.

Mice fed doxycycline from embryonic to adult stages were normal, as expected if the Smad7 transgene is silent and unable to block signaling. But a few weeks after doxycycline was removed from their food, the mice became diabetic: their blood insulin levels dropped and glucose accumulated to abnormally high levels. Looking inside the ß cells, the researchers found that Smad7 expression caused the near disappearance of MafA, a protein known to directly activate the insulin gene. Indeed, the pancreas of mice expressing Smad7 contained far less insulin than normal, confirming that Smad7 prevented insulin synthesis. Smad7 also prevented the expression of key markers of mature ß cell identity, such as Menin and p27 ^Kip1^, two proteins that prevent ß cell proliferation. Of note, when the mice were given doxycycline again, their diabetes disappeared in a few days, and expression of MafA, Menin, and p27 ^Kip1^ returned to normal. Therefore, Smad7 did not simply destroy the cells, but reversibly altered their ß cell identity.

Having thus firmly established the importance of TGF-ß signaling for the maintenance of ß cell characteristics, the researchers can now look for the precise TGF-ß molecules that act in mature ß cells, and continue piecing together the ß cell puzzle.

